# *Ocimum basilicum* and *Lagenaria siceraria* Loaded Lignin Nanoparticles as Versatile Antioxidant, Immune Modulatory, Anti-Efflux, and Antimicrobial Agents for Combating Multidrug-Resistant Bacteria and Fungi

**DOI:** 10.3390/antiox13070865

**Published:** 2024-07-19

**Authors:** Lamiaa A. El-Samahy, Yasmine H. Tartor, Adel Abdelkhalek, Ioan Pet, Mirela Ahmadi, Sameh M. El-Nabtity

**Affiliations:** 1 Department of Pharmacology, Faculty of Veterinary Medicine, Arish University, Arish 45511, Egypt; lamia.ahmed@vet.aru.edu.eg; 2 Department of Microbiology, Faculty of Veterinary Medicine, Zagazig University, Zagazig 44511, Egypt; 3 Faculty of Veterinary Medicine, Badr University in Cairo (BUC), Badr City 11829, Egypt; adel.abdelkhalek@buc.edu.eg; 4 Department of Biotechnology, Faculty of Bioengineering of Animal Resources, University of Life Sciences “King Mihai I” from Timisoara, 300645 Timisoara, Romania; ioanpet@usvt.ro; 5 Department of Pharmacology, Faculty of Veterinary Medicine, Zagazig University, Zagazig 44511, Egypt; smn@zu.edu.eg

**Keywords:** lignin, *Lagenaria siceraria*, *Ocimum basilicum*, *S.* Typhimurium, *T. rubrum*, anti-efflux activity, anti-dermatophytes activity, drug delivery

## Abstract

Lignin nanoparticles emerged as a promising alternative for drug delivery systems owing to their biodegradability and bioactive properties. This study investigated the antimicrobial activity of the ethanolic extract of *Ocimum basilicum-*loaded lignin nanoparticles (OB-LNPs) and *Lagenaria siceraria* seed oil-loaded lignin nanoparticles (LS-LNPs) to find a solution for antimicrobial resistance. OB-LNPs and LS-LNPs were tested for their antimicrobial potential against *Escherichia coli*, *Enterococcus faecalis*, *Klebsiella pneumoniae*, *Staphylococcus aureus*, *Salmonella enterica*, *Trichophyton mentagrophytes*, *Trichophyton rubrum*, and *Microsporum canis*. OB-LNPs and LS-LNPs were further tested for their anti-efflux activity against ciprofloxacin-resistant *Salmonella enterica* strains and for treating *Salmonella* infection in a rat model. We also investigated the antifungal efficacy of OB-LNPs and LS-LNPs for treating *T. rubrum* infection in a guinea pig model. Both OB-LNPs and LS-LNPs showed strong antimicrobial potential against *S*. Typhimurium and *T. rubrum* infections. LS-LNPs showed antibacterial activity against *Salmonella enterica* species with a MIC range of 0.5–4 µg/mL and antifungal activity against *T. rubrum* with a MIC range of 0.125–1 µg/mL. OB-LNPs showed antibacterial activity against *Salmonella enterica* species with a MIC range of 0.5–2 µg/mL and antifungal activity against *T. rubrum* with a MIC range of 0.25–2 µg/mL. OB-LNPs and LS-LNPs downregulated the expression of *ramA* and *acrB* efflux pump genes (fold change values ranged from 0.2989 to 0.5434; 0.4601 to 0.4730 for *ramA* and 0.3842–0.6199; 0.5035–0.8351 for *acrB*). Oral administration of OB-LNPs and LS-LNPs in combination with ciprofloxacin had a significant effect on all blood parameters, as well as on liver and kidney function parameters. Oxidative stress mediators, total antioxidant capacity, and malondialdehyde were abolished by oral administration of OB-LNPs and LS-LNPs (0.5 mL/rat once daily for 5 days). Interferon-γ and tumor necrosis factor-α were also reduced in comparison with the positive control group and the ciprofloxacin-treated group. Histopathological examination of the liver and intestine of OB-LNPs and LS-LNPs-treated rats revealed an elevation in *Salmonella* clearance. Treatment of *T. rubrum*-infected guinea pigs with OB-LNPs and LS-LNPs topically in combination with itraconazole resulted in a reduction in lesion scores, microscopy, and culture results. In conclusion, OB-LNPs and LS-LNPs possess immunomodulatory and antioxidant potential and can be used as naturally derived nanoparticles for drug delivery and treatment of Salmonellosis and dermatophytosis infections.

## 1. Introduction

Recently, nanotechnology has developed a correlation between the physical and biological sciences by applying nanostructures and nanophases in different fields of science, particularly nanomedicine and nano-based drug delivery systems [1]. Nanoparticles are small nanospheres designed at the atomic or molecular level [2]. Therefore, they can move more freely in the human body than larger materials [3]. A remarkable increase is observed in green technologies for the synthesis of nanomaterials from forest process streams such as lignin [4]. Lignin nanoparticles (LNPs) emerged from the recent valorization pathways described for lignocellulosic biomass in the field of biomedical applications [5]. Lignin is the most abundant amorphous aromatic biopolymer, so it has received great interest in recent years [5]. Among the available natural biodegradable drug carriers, LNPs have proven to be the most promising green material for drug delivery because of the low cost of the raw material. Moreover, lignin possesses heterogeneous chemical structures; it is simple and controllable to be converted into uniform NPs [6]. Lignin contains several active functional groups, including phenyl, aliphatic, carbonyl, methoxy, and phenolic hydroxyl groups that represent active sites for chemical modifications by oxidation, sulfonation, graft copolymerization, and hydroxymethylation reactions [7]. Owing to their antioxidant, antimicrobial, anticancer, and anti-inflammatory activities, LNPs have been used for drug delivery [8]. LNPs are used for stabilizing essential oils (Eos) and promoting their growth inhibition activity against pathogens, especially bacterial pathogens [9,10]. *Lagenaria siceraria* is cultivated in Egypt for its seeds, which are utilized for making salad oil [11]. *Lagenaria siceraria* seed oil contains a high amount of fatty acids and sterol compounds. Also, they are beneficial for health owing to their high content of polyunsaturated fatty acids such as linolenic and linoleic acids, which decrease the risk of cardiovascular and atherosclerosis diseases through their resistance to oxidation [12]. *Lagenaria siceraria* seed extract showed antibacterial and antifungal activities compared with those reported for fruits. These variations in activity are attributed to the plant part utilized, the source of the microbial strains, or environmental factors [13]. *L. siceraria* seed extract contains different phytochemical compounds such as alkaloids, cardiac glycosides, terpenoids, saponins, carbohydrates, phenols, and reducing sugars, which were considered biologically active with antimicrobial properties [14]. *Ocimum basilicum* is cultivated globally for its Eo, which is applied in medicine/pharmaceutical, cosmetics, perfumery, and as a flavoring agent [15]. *O. basilicum* has been used as a sedative, preservative, digestive regulator, and diuretic [16]. It has also been recommended for the treatment of headaches, coughs, infections of the upper respiratory tract, kidney malfunction, and toxin elimination [17]. The antioxidant and antimicrobial activities of *O. basilicum* are attributed to its content of phenolic and aromatic compounds such as phenolic acids and flavonol-glycosides [18]. Both *Ocimum* Eo and its extracts have antibacterial activity against Gram-positive and Gram-negative bacteria [19]. *O. basilicum* extract contains different chemical compounds, including camphene, α-pinene, β-pinene, limonene, myrcene, cis-ocimene, linalool, camphor, methyl chavicol, γ-terpineol, citronellol, geraniol, eugenol, methyl cinnamate, and other terpenes [18].

Salmonellosis is a globally distributed food-borne zoonotic disease caused by the consumption of contaminated poultry meat [20]. *Salmonella enterica* serovar Typhemurium is one of the most known causes of food poisoning due to the consumption of contaminated food or water [21]. Multidrug-resistant (MDR) *Salmonella* typically spreads because of antibiotic overuse, especially in the veterinary sector. Therefore, an integrative “One Health” approach for non-typhoidal *Salmonella* (NTS) surveillance among human, poultry, and animal populations is necessary to mitigate the potential risk to public health posed by the transmission of MDR NTS from poultry [22]. Recently, ciprofloxacin (CIP) and tigecycline (TIG) co-resistance were detected in extensive drug-resistant (XDR) *Salmonella enterica* isolates from humans and poultry. These isolates overexpress *ram*A [20]. Overexpression of the main efflux pump (*Acr*AB-*Tol*C) in *Salmonella* efflux systems leads to a reduction in cellular drug accumulation and MDR phenotype, including CIP and TIG [23,24].

Dermatophyte infections are one of the most prevalent fungal diseases globally, causing significant morbidity, especially in tropical areas, owing to the hot and moist climate [25,26]. One of the most widely distributed filamentous fungi is *Trichophyton rubrum*. It is the main cause of cutaneous mycosis and onychomycosis [27]. *T. rubrum* induces dermatophytosis through the adhesion of arthroconidia to the stratum corneum, after which keratin destruction occurs [28,29]. Dermatophytosis is accompanied by various symptoms such as redness, scaling, and itching in the affected region, along with mild to severe alopecia [30,31]. Several topical and systemic medications are used to treat dermatophytosis, such as itraconazole and terbinafine. These drugs need long-term adherence and can induce drug resistance and toxicity [32,33].

Due to the pharmacological properties of the two extracts and to lignin itself, the current study is carried out to (i) evaluate the antimicrobial potentials of *L*. *siceraria* seeds extract loaded lignin nanoparticles (LS-LNPs) and *O*. *basilicum* loaded lignin nanoparticles (OB-LNPs) against different Gram-positive, Gram-negative bacteria, and dermatophytes, (ii) develop an alternative naturally derived LS-LNPs and OB-LNPs as a drug delivery system to overcome antimicrobial resistance in *Salmonella enterica* strains, (iii) evaluate their antimicrobial potential against salmonellosis in a rat model, and (iv) evaluate their anti-dermatophyte activity in vitro and in vivo in a guinea pig infection model.

## 2. Materials and Methods

### 2.1. Bacterial and Fungal Strains

To investigate the antimicrobial activity of LS-LNPs and OB-LNPs, a total of eighty bacterial and dermatophytes strains (10 of each species) were included in this study, including *Escherichia coli*, *Enterococcus faecalis*, *Klebsiella pneumoniae*, *Staphylococcus aureus*, *Salmonella enterica*, *Trichophyton mentagrophytes*, *T. rubrum*, and *Microsporum canis*. These strains were identified using the primers listed in Table S1. The source of each strain is listed in Table S2. The commercially available antisera (Denka Seiken Co., Ltd., Coventry, UK) was used for serotyping *Salmonella* isolates.

### 2.2. Antimicrobial Susceptibility of Tested Strains

The disc diffusion method was performed for the determination of the antibiogram of each strain according to the Clinical and Laboratory Standards Institute (CLSI) [34]. Mueller Hinton agar plates (MHA) (Merk, Darmstadt, Germany) were inoculated with the inoculum suspension of each bacterial strain containing (1 × 10^8^ CFU/mL) then the antimicrobial discs (Oxoid, Hampshire, UK) including chloramphenicol (30 µg), ciprofloxacin (5 µg), tigecycline (15 µg), vancomycin (30 µg) cefoxitin (30 µg), ampicillin (10 µg), gentamycin (10 µg), fosfomycin (50 µg), sulfamethoxazole-trimethoprim (23.75/1.25 µg), colistin (10 µg), imepinim (10 µg) and ceftriaxone (30 µg), aztreonam (30 µg), tetracycline (30 µg), amikacin (30 µg), tobramycin (10 µg), nalidixic acid (30 µg), meropenem (10 µg), ertapenem (10 µg), cefazolin (30 µg), cefuroxime (30 µg), cefepime (30 µg), ceftriaxone (30 µg), amoxicillin-clavulanic acid (20/10 µg), ampicillin-sulbactam (20/10 µg) doxycycline (30 µg), rifampin (30 µg), erythromycin (15 µg) piperacillin/tazobactam (20/10 µg), aztreonam (30 µg) fusidic acid (10 µg), streptomycin, (10 µg) penicillin (30 µg) were inoculated and incubated at 37 °C for 24 h. The inhibition zone diameters were measured and interpreted according to CLSI. Disc diffusion results were confirmed by measuring the minimum inhibitory concentration (MIC) for colistin, ciprofloxacin, tigecycline, and vancomycin using the broth microdilution method [34]. CLSI determined the breakpoints for ciprofloxacin, tigecycline, colistin, and vancomycin as ≥1 µg/mL, >2 µg/mL, ≥4 µg/mL, and >8 µg/mL, respectively [34].

The antifungal susceptibility of dermatophyte species was tested using a broth microdilution assay [35]. Serial two-fold dilutions of antifungals were prepared to reach the final concentration of 0.25–128 µg/mL for fluconazole and 0.03–16 µg/mL for itraconazole, voriconazole, ketoconazole, miconazole, and terbinafine. Fungal suspensions (2 × 10^3^ CFUs/mL) were prepared in RPMI 1640 medium (Life Technologies, New York, NY, USA) and added to a 96-well microtitre plate (Iwaki, Tokyo, Japan). The plates were incubated at 28 °C for 72 h.

### 2.3. Preparation of Ocimum basilicum Ethanolic Extract and *Lagenaria siceraria* Sedd Oil

*Ocimum basilicum* leaves that were harvested from a garden in Egypt and authenticated by a taxonomist from the Department of Botany, Faculty of Science, Zagazig University, Egypt, were subjected to extraction using 95% ethyl alcohol (Sigma, St. Louis, MO, USA). Using the Soxhlet extractor apparatus (Electrothermal, London, UK), 50 g of plant leaf powder was put inside the thumble, and 500 mL of 95% ethyl alcohol was put in the flask. A clear, colorless solvent appeared in the extracting unit after the extraction was completed for 24 h at a temperature that was maintained at 50 to 60 °C. Then, the extract was dried at 40–50 °C in an oven (Thermo Fisher Scientific Inc., Tokyo, Japan). The dry extract was kept in an incubator set at 40 °C to ensure total dryness. The extract was kept at −20 °C until use [36].

The fruits of *Lagenaria siceraria* were collected and washed, then the seeds were exposed, gently collected, washed, and dried for 72 h in the shade. After that, the seeds were pulverized with a husk, and the pulverized seeds (804.40 g) were soaked in 1.2 L of dichloromethane (Sigma-Aldrich, St. Louis, MO, USA) for 72 h and agitated frequently at room temperature. The dry residue was then soaked in methanol for 72 h with frequent agitation, filtered, and concentrated [14].

### 2.4. Synthesis and Characterization of LS-LNPs and OB-LNPs

**Figure 1:**
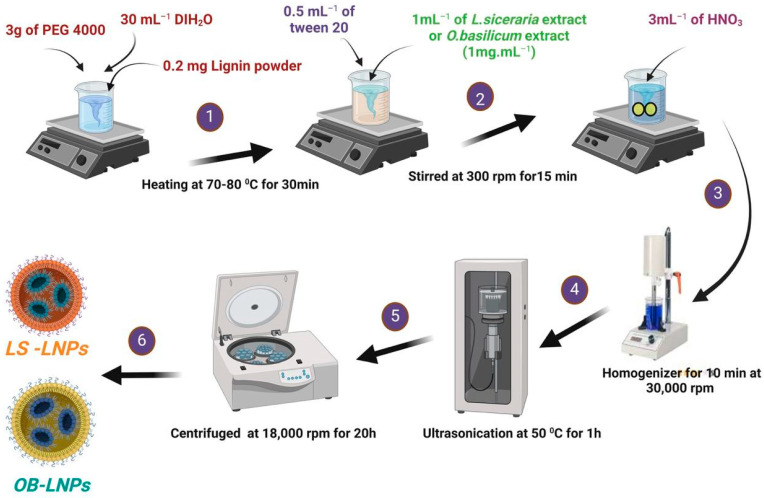
Schematic illustration of the green synthesis of LS-LNPs and OB-LNPs.

LS-LNPs and OB-LNPs were prepared as previously described [37], with some modifications. As presented in Figure 1, lignin (0.2 mg) (Green Value S.A, Orbe, Switzerland) was dissolved in 30 mL of polyethylene glycol 4000 (PEG) (Oxoid, Hampshire, UK), 0.5 mL of tween 20 (Merck, Darmstadt, Germany) was added, and the mixture was stirred at 300 rpm for 15 min at 50 °C to preactivate lignin. Then 1 mL of *L. siceraria* seed oil and *O. basilicum* ethanolic extract at a final concentration of 1 mg/mL was added. After that, 3 mL of nitric acid (HNO_3_) (Oxoid, Hampshire, UK) was added drop by drop, and the solution was subjected to homogenizer (Heidolph, DIAX 900, Schwbach, Germany) for 10 min at 30,000 rpm, then ultrasound (20 kHZ, 50% amplitude, Ti horn) for 1 h at 50 °C to produce LS-LNPs and OB-LNPs (VCX 750 ultrasonic processor, Sonics & Materials, Inc., Newtown, CT, USA). The mixture was centrifuged at 18,000 rpm for 20 min to remove unreacted molecules in the supernatant. The pellet was resuspended in distilled water and concentrated five times, and the particles were disaggregated by applying a low-intensity ultrasound. Finally, the NPs were centrifuged at 500× *g* for 10 min to remove larger aggregates. The LS-LNPs and OB-LNPs were stored at 4 °C. Preactivation of bulk lignin nanoparticles (LNPs) without *L. siceraria* and *O. basilicum* was carried out as described above. Transmission electron microscopy (TEM) and selected area electron diffraction (SAED) were used to study the morphology and distribution of the NPs by placing 10 μL of diluted sample onto holey carbon films on copper grids [38]. The samples were observed using a JEOL JEM-2100 LaB6 microscope (JEOL Ltd., Tokyo, Japan) operating at an accelerating voltage of 200 kV. The UV-vis spectra were obtained using a GENESYS™ G10S spectrophotometer (Thermo Fisher Scientific, Waltham, MA, USA). The absorbance of the solutions was measured in the range of 200–800 nm. The Zeta potential of the particles was determined using a Zetasizer Nano ZS™ ZEN3600 (Malvern Instruments Ltd., Malvern, UK). Carl Zeiss Microscopy (GmbH, Rudolf Eber Str. Oberkochen, Germany) was used for obtaining X-ray diffractograms (XRD) of the nanoparticles. A Fourier transform infrared (FTIR) spectrometer (JASCO FT-IR 4100 spectrometer, Hachioji, Tokyo, Japan) was used for obtaining the FTIR spectra over a range of 4000–500 cm^−1^.

### 2.5. Testing Antimicrobial Activity of LS-LNPs and OB-LNPs

The antimicrobial activities of LS-LNPs and OB-LNPs were evaluated against bacterial and fungal strains by agar-well diffusion and broth microdilution tests. Before testing, bacterial strains were subcultured on bile esculin agar (Oxoid Ltd., Hampshire, UK) for *E. faecalis*, Eosin Methylene Blue (EMB) (Oxoid Ltd., Hampshire, UK) for *E. coli* and *K. pneumoniae*, Baired Parker agar with egg yolk tellurite emulsion (Oxoid Ltd., Hampshire, UK) for *S. aureus,* and Xylose Lysine Desoxycolate (XLD) (Oxoid Ltd., Hampshire, UK) for *Salmonella enterica* and incubated at 37 °C for 24 h. Dermatophyte species were subcultured on mycobiotic agar medium (CONDA, Madrid, Spain) slants and incubated at 28 °C for 7 days.

#### 2.5.1. Agar Well Diffusion Test

Suspensions of each bacterial and fungal strain were prepared in sterile saline (1.5 × 10^8^ CFU/mL) and then swabbed on MHA (Oxoid Ltd., Hampshire, UK). Wells (8 mm) were cut into each inoculated agar plate, and a 100 µL aliquot of LS-LNPs and OB-LNPs was pipetted into each well. MHA plates were incubated at 37 °C for 24 h and at 25 °C for 96 h, with daily examination of bacteria and dermatophytes, respectively. The inhibition zone diameters were measured and interpreted [39].

#### 2.5.2. Broth Microdilution Test

Using 96-well plates (Iwaki, Tokyo, Japan), serial two-fold dilutions of LS-LNPs and OB-LNPs (0.125 µg/mL to 512 µg/mL) were prepared in Mueller Hinton broth (BD Difco, Thermo Fisher Scientific Inc., Tokyo, Japan) or RPMI 1640 media supplemented with 3-(N-morpholino) propanesulfonic acid (Sigma-Aldrich, St. Louis, MO, USA), for testing bacteria and fungi, respectively. Each well was inoculated with 100 µL of the suspension of standardized inoculum (10^6^ CFU/mL), then incubated at 37 °C for 24 h and at 28 °C for 48 h with daily examination of bacteria and dermatophytes, respectively. The test was performed in triplicate, and a negative control broth and a growth control (broth and inoculum) were included. MIC was determined as the lowest concentration of antimicrobial agents that inhibits the growth of bacteria and fungi [40,41].

### 2.6. Testing of the Antiefflux Activity of LS-LNPs and OB-LNPs on Salmonella enterica Species

The efflux pump activity of 10 ciprofloxacin-resistant and intermediate *Salmonella enterica* strains was determined using the ethidium bromide (EtBr) cartwheel method [42]. Briefly, tryptic soy agar (TSA, Oxoid, UK) plates containing EtBr (Sigma-Aldrich, Hamburg, Germany) at concentrations ranging from 0 to 2 mg/L were prepared on the same day of the experiment and protected from light. The plates were then divided into 10 sectors using radial lines (cartwheel pattern). O.D-adjusted cultures (0.6 at 600 nm) were swabbed onto EtBr-agar plates. Each plate included a reference strain that served as a comparative control. The swabbed EtBr agar plates were wrapped in aluminum foil, incubated at 37 °C for 18 h and examined under an UV transilluminator (Accuris Instruments, New York, NY, USA). The minimum concentration of EtBr that produced fluorescence in the bacterial mass was recorded. The index for efflux activity of the ciprofloxacin-resistant *Salmonella* strains was calculated relative to the reference strain according to the following formula:

Efflux activity index = MCEtBr (MDR) − MCEtBr (REF)/MCEtBr (REF).

where MCEtBr (MDR) and MCEtBr (REF) represent the minimum concentration of EtBr that produces fluorescence in the colonies of MDR-tested bacteria and the reference strain. Strains expressing increased levels of efflux activity by the EtBr-agar cartwheel method were tested in the presence of LS-LNPs and OB-LNPs. A significant efflux inhibition activity was considered when a ≥4-fold decrease in the MIC values was detected [43,44].

### 2.7. Quantification of the Transcription Levels of Efflux Pump Genes

SYBR green real-time PCR was used to determine the relative expression levels of the *ram*A and *acr*B efflux genes of the strains having detectable efflux pump activity. Total RNA was extracted from *Salmonella* strains using the QIAamp RNeasy Mini kit (Qiagen, Hilden, Germany) following the manufacturer’s instructions. The relative quantification was done in triplicate using the Quanti Tect SYBR green real-time PCR kit (Qiagen, Germany) in an MX3005P real-time PCR thermal cycler (Agilent, La Jolla, CA, USA) following the manufacturer’s recommendations. Melting curve analysis was performed to confirm the specificity of the assays. The 16S rRNA housekeeping gene was used as a normalizer [45], and fold change values were estimated using the 2^−∆∆CT^ method [46]. A susceptible *Salmonella* Virchow isolate was used as a comparative control.

### 2.8. In Vivo Testing of Antibacterial Activities of LS-LNPs and OB-LNPs

Seventy Albino rats (150–180 g) were obtained from the laboratory animal farm at the Faculty of Veterinary Medicine, Zagazig University. Rats were maintained on a standard pellet diet and tap water ad libitum and were kept in plastic cages under a 12 h light/dark cycle at a temperature of 22–24 °C. Rats were acclimatized to the environment for two weeks prior to experimental use. Rats were randomly divided into seven groups (10 rats per group). Rats were fasted overnight and administered 1 mL of a saline solution containing 1 × 10^6^ CFU/mL of *S.* Typhimurium intraperitoneally [47]. Animals in group 1 (G1) were not infected and received 1 mL of sterile saline. G2 were only infected. G3 were infected and treated with 45 mg/kg ciprofloxacin (CIP), G4 were treated with CIP + OB-LNPs (2.5 mL/kg bw: 0.5 mL orally daily for 5 days), G5 were treated with CIP + LS-LNPs (2.5 mL/kg bw: 0.5 mL orally daily for 5 days), and G6 and G7 received OB-LNPs and LS-LNPs, respectively. The study protocol was approved by the Institutional Animal Care and Use Committee, Zagazig University, Egypt (approval number ZU-IACUC/2/F/114/2021).

#### 2.8.1. Measurement of Bacterial Load in Organs

On the 3rd, 7th, and 14th days post-challenge, the spleen, liver, and intestine were aseptically collected, and 1 gm of organs was homogenized in 1 mL of sterile saline using a tissue homogenizer at 4 °C. Appropriate 10-fold dilutions were spread-plated on XLD agar medium (Oxoid Ltd., Hampshire, UK) and incubated at 37 °C for 24 h. Colonies were counted, and the total CFU was calculated [48].

#### 2.8.2. Blood Biochemical and Immunological Parameters

At 7- and 14-days post-challenge, five rats from each group were randomly chosen for the aseptic collection of blood samples from the tail vein. The collected blood was divided into two equal parts: the first part was collected on EDTA (Oxoid, UK) to determine Hb and differential WBCs counts. The second part was immediately centrifuged at 3500 rpm for 15 min, and the serum was used to measure biochemical biomarkers for the liver (ALT, AST, albumin, and total protein) and kidney functions (urea and creatinine). The phagocytosis activity of neutrophils and monocytes was measured, and the phagocytic index (PI), the average number of ingested particles/cells, was calculated. Evaluation of mediators of oxidative stress: Total antioxidant capacity (TAC) and malondialdehyde (MDA) were analyzed using commercial kits (Jiancheng Biotechnology Institute, Nanjing, China). Serum pro-inflammatory cytokines interferon gamma (IFN-γ) and tumor necrosis factor alpha (TNF-α) were analyzed spectrophotometrically using enzyme-linked immunosorbent assay (ELISA) kits (Cusabio Biotech Co. Ltd., Wuhan, China).

#### 2.8.3. Histopathological Examination of Internal Organs

On the 7th day post-challenge, liver and intestine specimens were preserved in a 10% formalin solution. The samples were then dehydrated in gradual ethanol (70–100%) and cleared in xylene (Oxoid, UK). Samples were then embedded in paraffin wax to prepare sections (5 µm thickness), which were subsequently stained with hematoxylin and eosin (H&E).

### 2.9. In Vivo Testing of the Anti-Dermatophyte Activity of LS-LNPs and OB-LNPs

Thirty-five guinea pigs, weighing approximately 250–300 g were housed in adequate circumstances for five days. The animals were randomly assigned to seven groups.

*T. rubrum* isolate was subcultured on mycobiotic agar slants and incubated at 30 °C for 7 days. A suspension of conidia in sterile saline was prepared at 1 × 10^8^ conidia/mL for animal inoculation. An area of about 2.5 cm × 2.5 cm on the back of the animal was clipped and shaved, then gently abraded with a sterile fine grit sandpaper. The conidial suspension was applied and rubbed thoroughly on the abraded skin [26]. To validate the success of the infection model, the inoculated areas were disinfected with 70% ethyl alcohol, and skin scrapings and hair samples were collected using a sterile toothbrush for subsequent microscopic examination using 20% KOH. The animals were clinically evaluated using lesion scores twice weekly for a 2-week period in the treatment study [49]. The infected animals in group 1 (G1) were (non-infected control negative group), G2 were infected and untreated (control positive group), G3 were infected and treated with 10 mg/kg itraconazole daily by oral gavage for 12 days, G4 were infected and treated with LS-LNPs topically + Itra, G5 were infected and treated with OB-LNPs topically + Itra, G6 were treated with OB-LNPs, and G7 received LS-LNPs. On days 3, 7, 10, and 14 post-treatment (PT), redness, scales, and alopecia were scored as previously described [50]. Scales and hair samples were collected on days 3, 7, 10, 14, and 28 PT for microscopic examination and culturing on mycobiotic agar medium (CONDA, Madrid, Spain) and dermatophyte test medium (DTM) plus dermatophyte supplement (Himedia, Mumbai, India). The cultures were incubated at 25 °C for a week and observed daily. Animals were kept under observation for up to 28 days PT to evaluate the final clinical cure rate.

### 2.10. Data Analysis

The data were processed using Microsoft Excel (v16.0, Microsoft Corporation, Redmond, WA, USA). Levene and Shapiro–Wilk tests were performed to assess the normality and homogeneity of variance [51]. Data analysis was conducted using a general linear model in the statistical analysis system [52] to determine the significant effects of different treatments on blood parameters. Multiple comparisons among means were conducted using Duncan’s multiple range test. The exact Wilcoxon test was used for comparisons between untreated and treated animals on specific days (3, 7, 10, and 14). Graphs were generated using GraphPad Prism software 9.0 (GraphPad, San Diego, CA, USA). Statistical significance was defined as a *p*-value less than 0.05.

## 3. Results

### 3.1. Physicochemical Properties and Transmission Electron Microscopy of LS-LNPs and OB-LNPs

**Figure 2:**
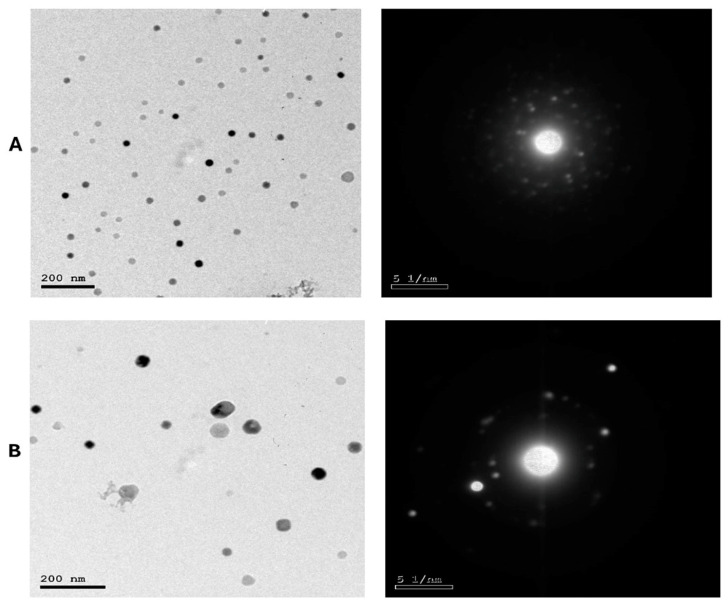
TEM micrographs and selected area electron diffraction (SAED) patterns of LS-LNPs (**A**) and OB-LNPs (**B**) showing nano sphere shape with concentric rings of brilliant spots.

**Figure 3:**
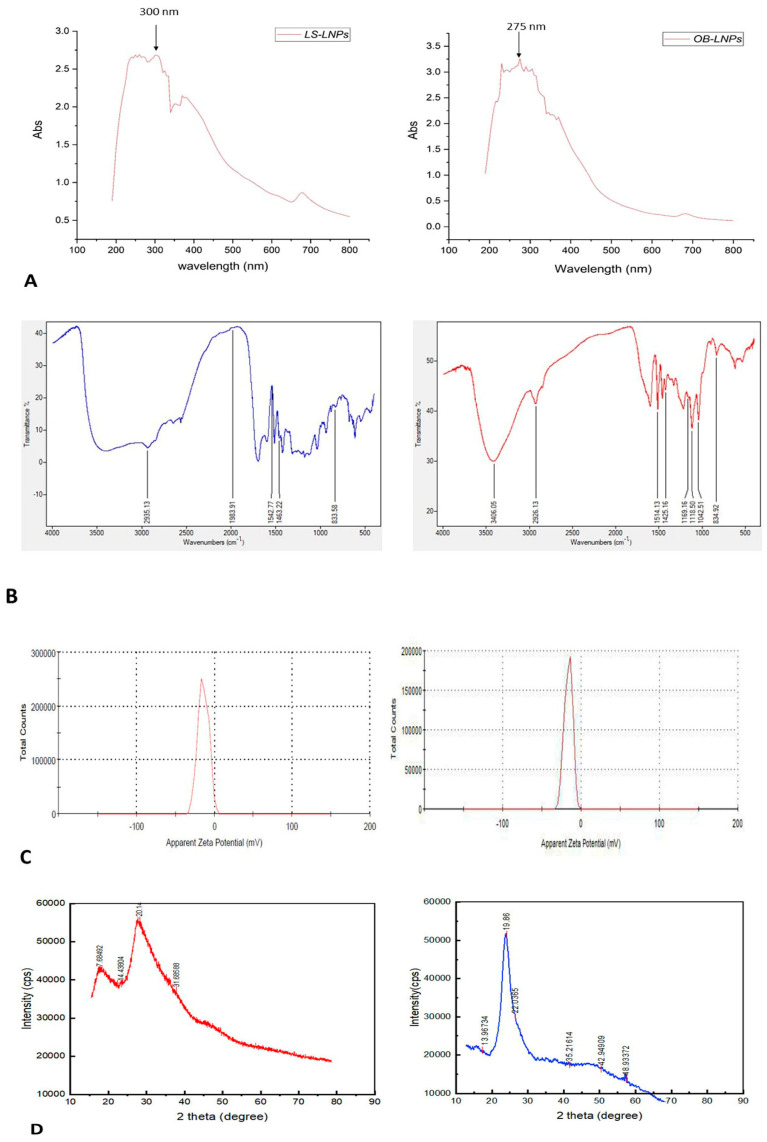
UV-visible spectra (**A**), FT-IR spectra (**B**), zeta potential (**C**), and XRD patterns (**D**) of LS-LNPs and OB-LNPs.

As presented in Figure 2A,B, LS-LNPs and OB-LNPs vesicles are spherical in shape and homogenous in size with no agglomeration. The UV–vis spectra show characteristic surface plasmon resonance bands, with maximum absorption bands detected at 300 and 275 nm for LS-LNPs and OB-LNPs, respectively (Figure 3A). 

As revealed in Figure 3B, The FTIR absorption peak at 2935.18 cm^−1^ is assigned to O-H stretching in the carboxylic acids of *L. siceraria* oil. The absorption peak at 1976.03 cm^−1^ represented X=C=Y of alkene and ketene groups. The C=O conjugation of the carboxylic group with phenyl is represented by a peak at 1542.88 cm^−1^. The peak at 1463 cm^−1^ belongs to the methyl scissoring vibrations of proteins. The peak at 833.58 cm^−1^ indicated C-H deformation ring vibrations and C-OH out of plane bending (C-H out of plane in positions 2 and 6 of S units and in all positions of H units of lignin). These spectral peaks indicate the presence of polyphenols, carbonyl, and alcoholic functional groups. 

The FTIR spectra of OB-LNPs (Figure 3B) revealed a strong absorption peak at 3389 cm^−1^ corresponding to phenols and alcohols with a free O-H group in lignin. Also, C-O vibrations in conjugated ester groups of lignin were indicated with an absorption peak at 1169.16 cm^−1^. The spectrum at 1425.16 cm^−1^ was assigned to the C=O carbonyl group stretching of *O. basilicum* extract. In addition, another spectral around 1118.5 cm^−1^ corresponds to the C-O in the plane bending of alkenes, alcohols, and carboxylic acids. The band at 2933.37 cm^−1^ indicated the presence of alkyl and CHO groups. Both the 1514 and 1042.5 cm^−1^ peaks indicated vibration stretching for the C=O carbonyl group. Methylene scissoring vibrations of proteins correspond to the spectrum at 1463 cm^−1^.

The zeta potential values of LS-LNPs and OB-LNPs were −14.3 mV and −17.0 mV (Figure 3C), respectively. The XRD patterns of the LS-LNPs and OB-LNPs are illustrated in Figure 3D. Three distinct diffraction peaks were observed at 2θ = 19.68°, 29.64°, and 41.81°, corresponding to the 2945, 3002, and 3095 crystalline planes of LS-LNPs, respectively. The distinct diffraction peaks in OB-LNPs were detected at 2θ = 19.58°, 48.76°, and 25.46°, which corresponded with the 531, 575, and 537 crystalline planes of OB-LNPs, respectively.

### 3.2. The Antimicrobial Activity of LS-LNPs and OB-LNPs

Preliminary screening of the antimicrobial activity of LS-LNPs and OB-LNPs indicated that LS-LNPs and OB-LNPs have strong antimicrobial potential against *Salmonella enterica* species and *T. rubrum* (Table 1). LS-LNPs showed antibacterial activity against *Salmonella enterica* species with a MIC range of 0.5–4 µg/mL and antifungal activity against *T. rubrum* with a MIC range of 0.125–1 µg/mL. OB-LNPs showed antibacterial activity against *Salmonella enterica* species with a MIC range of 0.5–2 µg/mL and antifungal activity against *T. rubrum* with a MIC range of 0.25–2 µg/mL. While the antimicrobial effect of both LS-LNPs and OB-LNPs was moderate against *S. aureus*, *E. faecalis*, *K. pneumoniae*, *E. coli*, *T. mentagrophytes*, and *M. canis.* LS-LNPs showed antibacterial activity against bacterial strains with MIC ranges of 4–16 µg/mL and 2–16 µg/mL for fungal isolates. OB-LNPs showed antibacterial activity against bacterial strains with MIC ranges of 2–16 µg/mL and 4–32 µg/mL for fungal isolates (Table S2). The antibacterial and antifungal activities of both LS-LNPs and OB-LNPs were confirmed using an in vivo model.

### 3.3. ramA and acrB Efflux Pump Genes Expression in Ciprofloxacin-Resistant Salmonella enterica Strains

**Figure 4:**
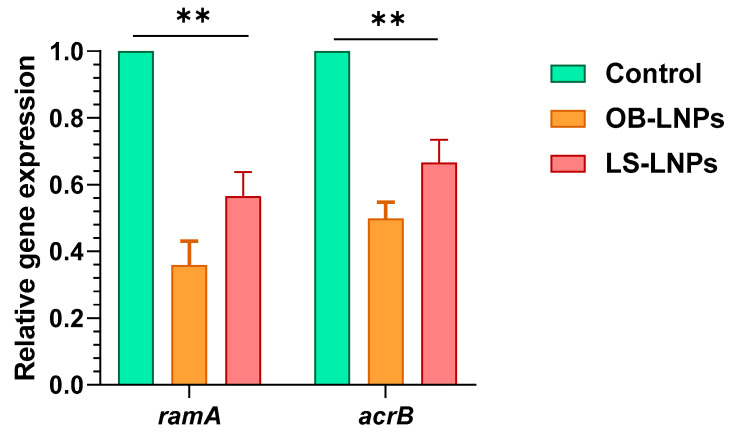
Expression of *ram*A and *acr*B efflux genes in *Salmonella enterica* strains treated with OB-LNPs and LS-LNPs. The transcription of the *ram*A and *acr*B genes significantly reduced in response to treatment with OB-LNPs and LS-LNPs, minimizing the OB-LNPs. ** indicates a highly significant difference at *p* value < 0.001.

Four ciprofloxacin-resistant *Salmonella* strains had efflux activity where they could pump EtBr outward (Table S3). The relative expression of the *ram*A and *acr*B efflux pump genes was studied at sub-MIC concentrations of LS-LNPs and OB-LNPs in ciprofloxacin-resistant isolates using qRT-PCR. After treatment of strains with sub-MIC concentrations of the nanocomposites, expression of the *ram*A and *acr*B efflux pump genes was downregulated, indicating an inhibitory effect on the efflux pump (Figure 4 and Table S3).

### 3.4. Biochemical, Haematological, Antioxidant, and Immunological Effects of CIP+LS-LNPs and CIP+OB-LNPs on Blood and Serum Constituents of S. *Typhimurium* Challenged Rats

The in vitro results were encouraging for in vivo testing of the antibacterial activity of LS-LNPs and OB-LNPs using *S*. Typhimurium infection model in rats. Biochemical analysis of the blood serum constituents showed that the challenge with *S*. Typhimurium had adverse effects on liver and kidney function parameters, represented by a significant increase (*p* < 0.05) in alanine aminotransferase (ALT), aspartate aminotransferase (AST), lactate dehydrogenase (LDH), urea, and creatinine levels at both 7- and 14-days post-challenge. Moreover, total protein (TP) and albumin (Alb) levels significantly decreased at both 7- and 14-days post-challenge. Serum ALT, AST, and LDH levels were significantly decreased in the CIP+LS-LNPs group at both 7- and 14-days post-challenge. TP and Alb levels in all treated groups were not significantly different from the negative control (NC) group. At 7 days post-challenge, there were no significant differences observed in urea levels between the CIP-, CIP+LS-LNPS-, and CIP+OB-LNPS-treated groups, as well as the NC group. However, at 14 days post-challenge, significant differences were found between the NC group and both the CIP+LS-LNPS- and CIP+OB-LNPS-treated groups. Regarding the level of creatinine, significant differences were observed between all treated groups and the NC group at both 7 and 14 days, except for the LS-LNPs-treated group and the NC group (Table 2).

Herein, the antioxidant capacity showed a significant increase in malondialdehyde (MDA) in the infected non-treated group in both 7- and 14-days post-challenge, respectively. The CIP+LS-LNPs treated group revealed non-significant differences from the NC group in both 7- and 14-days post-challenge. Total antioxidant capacity (TAC) indicated a significant decrease in the challenged group. CIP+LS-LNPS, CIP+OB-LNPS, LS-LNPS, and OB-LNPS-treated groups showed no significant differences from NC at 14th day post-challenge (Table 2).

The blood hemoglobin (Hb) concentration level was significantly reduced in the infected group, and a significant elevation was observed in all treated groups in both 7- and 14-days post-challenge. CIP+LS-LNPS, CIP+OB-LNPS, and OB-LNPS treated groups were not significantly different from NC at 14th day post-challenge. Regarding the immunity status, total leukocytic count (WBCs) and neutrophils were significantly elevated in the infected group for WBCs and for neutrophils in both 7- and 14-days post-challenge. The total leukocytic count (WBCs) and neutrophils of the CIP+ LS-LNPs treated group were not significantly different from the NC group at both 7- and 14-days post-challenge. In contrast, lymphocytes, phagocytosis, and phagocytic index showed a significant decrease in the challenged group at both 7- and 14-days post-challenge. A significant elevation in lymphocytes, phagocytosis, and phagocytic index was observed in the CIP+LS-LNPs treated group. The results of proinflammatory cytokines revealed a significant increase in the blood serum levels of IFN-γ and TNF-α in *S*. Typhimurium challenged group. Meanwhile, a significant reduction was detected in all treated groups in both 7- and 14-days post-challenge. The CIP+ LS-LNPs treated group revealed no significant differences in the blood serum levels of IFN-γ and TNF-α when compared with the NC group in both 7- and 14-days post-challenge (Table 2).

### 3.5. Bacterial Load Counting

**Figure 5:**
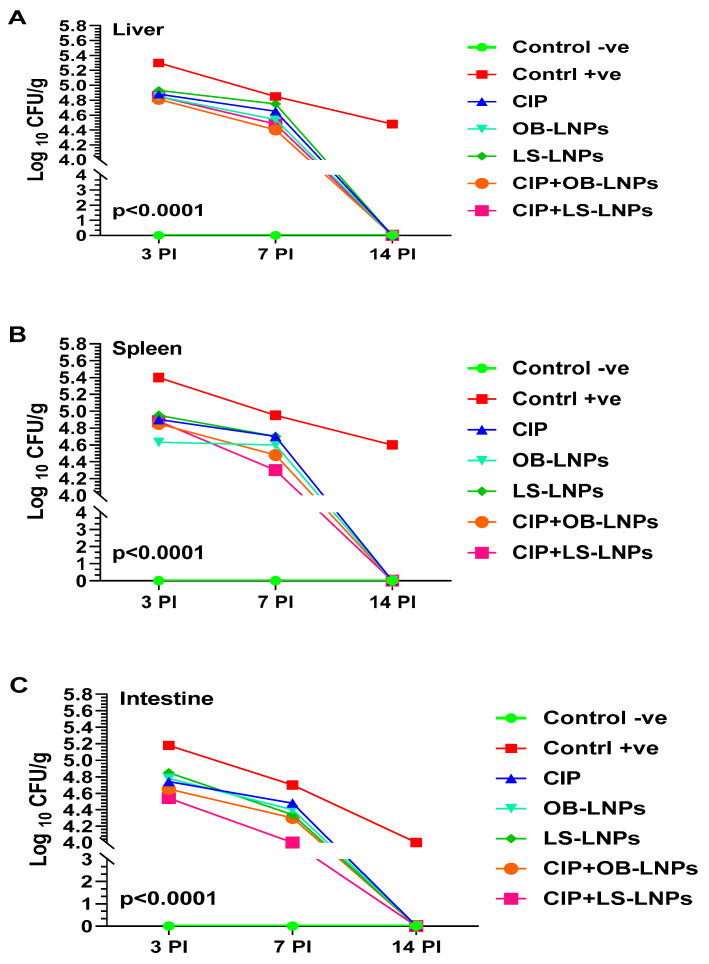
Total bacterial counts (Log_10_ CFU) in liver (**A**), spleen (**B**), and intestine (**C**) samples from rats in different groups treated with CIP (45 mg/kg bw), Cip+OB-LNPs (45 mg/kg bw + 2.5 mL/kg bw), Cip+LS-LNPs (45 mg/kg bw + 2.5 mL/kg bw), OB-LNPs (2.5 mL/kg bw), and LS-LNPs (2.5 mL/kg bw), daily for 5 days post *S*. Typhimurium challenge.

As depicted in Figure 5, liver, spleen, and intestinal homogenates revealed a significant decrease in *S*. Typhimurium counts at 3-, 7-, and 14-days post-challenge in the treated groups when compared with the non-treated group (*p* < 0.05). Whereas at 3 days post-challenge, bacterial counts in liver, spleen, and intestinal homogenates of the treated groups ranged between 4.5 log_10_ and 4.9 log_10_ CFU/g, compared with 5.2 log_10_ to 5.4 log_10_ CFU/g of the nontreated group. At 7 days post-challenge, *S.* Typhimurium counts in the liver, spleen, and intestinal homogenate of the treated groups ranged between 4 log_10_ and 4.7 log_10_ CFU/g when compared with 4.7 log_10_ to 5 log_10_ CFU/g in the non-treated group. Interestingly, at 14 days post-challenge, *S.* Typhimurium was not detected in the examined organs of the treated groups, but the bacterial load in the nontreated group was 4 log_10_ to 4.6 log_10_ CFU/g. The CIP+LS-LNPs group achieved the most prominent results among all treated groups (at day 7 post-challenge, log_10_ CFU/g of liver, spleen, and intestine of animals were 4.5, 4.3, and 4, respectively).

### 3.6. Histopathological Features of Intestinal and Hepatic Tissues Post S. *Typhimurium* Infection

**Figure 6:**
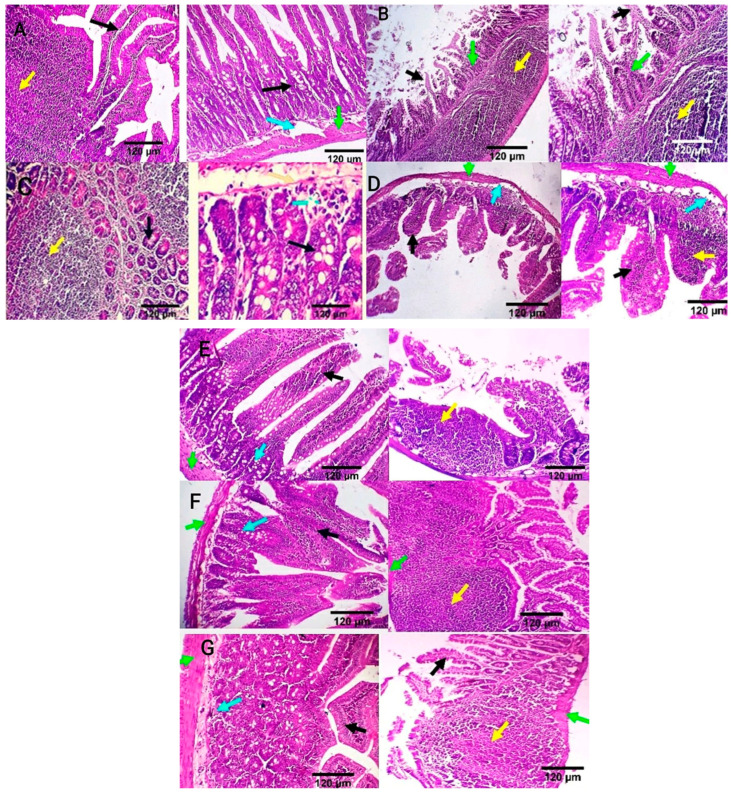
Photomicrograph from the ileum and jejunum of rats using H&E staining at 7th day post infection with *S*. Typhimurium. (**A**) noninfected and nontreated rats showing normal mucosal villi comprising absorptive columnar epithelium with variable number of goblet cells (black arrow), normal Peyer’s patch (yellow arrow), normal submucosa (light blue arrow), and muscular coat (green arrow). (**B**) infected, non-treated rats showing villous necrosis (black arrows), mucosal leucocytic infiltration (green arrows), and moderate lymphoid hyperplasia of Peyer’s patch (yellow arrows). (**C**) rats were infected and treated with 45 mg/kg CIP (ciprofloxacin) orally once daily for 5 days. The ileum revealed normal submucosal glands (black arrow) and mildly reactive Peyer’s patch (yellow arrow). The jejunum revealed normal jejunal mucosal villi comprising absorptive columnar epithelium with a variable number of goblet cells (black arrow), and few lymphocytes are seen in the submucosa (light blue arrow). (**D**) rats were infected and received CIP+OB-LNPs (45 mg/kg bw + 2.5 mL/kg bw). The jejunum and ileum show normal mucosal villi comprising absorptive columnar epithelium with a variable number of goblet cells, occasional lymphocytic infiltration (black arrow), and normal Peyer’s patch (yellow arrow), beside normal submucosa (light blue arrows) and muscular coat (green arrow). (**E**) rats were infected and treated with CIP+LS-LNPs (45 mg/kg bw + 2.5 mL/kg bw) showing normal mucosal villi comprising absorptive columnar epithelium with variable number of goblet cells (black arrow) and normal Peyer’s patch (yellow arrow), beside normal submucosa (light blue arrows) and muscular coat (green arrow). Ileal villi of some sections appear moderately stunted (white stars). (**F**) rats were infected and treated with OB-LNPs (2.5 mL/kg bw), showing normal mucosal villi comprising absorptive columnar epithelium with a variable number of goblet cells and occasional epithelial stratification (black arrow), moderately reactive Peyer’s patch (yellow arrow), normal submucosa (light blue arrows), and muscular coat (green arrow). (**G**) rats were infected and treated with LS-LNPs (2.5 mL/kg bw) daily for 5 days, showing normal mucosal villi with variable number of goblet cells (black arrow), normal Peyer’s patch (yellow arrow), normal submucosa (light blue arrows), and muscular coat (green arrow).

Intestinal tissue of the negative control group showed normal mucosal villi comprising absorptive columnar epithelium with a variable number of goblet cells and normal Peyer’s patches, submucosa, and muscular coat (Figure 6A). Meanwhile, in the positive control group, intestinal sections showed villous necrosis, mucosal leucocytic infiltration, and moderate lymphoid hyperplasia of Peyer’s patch. Villous atrophy with goblet cell metaplasia was also detected (Figure 6B). The ciprofloxacin-treated group displayed normal jejunal mucosa, normal submucosal glands, and mildly reactive Peyer’s patch. A few lymphocytes are seen in the submucosa (Figure 6C). CIP+OB-LNPs and CIP+LS-LNPs-treated groups showed a variable number of goblet cells and a moderately reactive Peyer’s patch. In addition, normal jejunal mucosal villi and a few lymphocytes are seen in the villous mucosa (Figure 6D,E). Both the OB-LNPs and LS-LNPs-treated groups revealed normal villi, comprising a variable number of goblet cells and a mildly reactive Peyer’s patch. The submucosa and muscular coat are apparently normal (Figure 6F,G).

**Figure 7:**
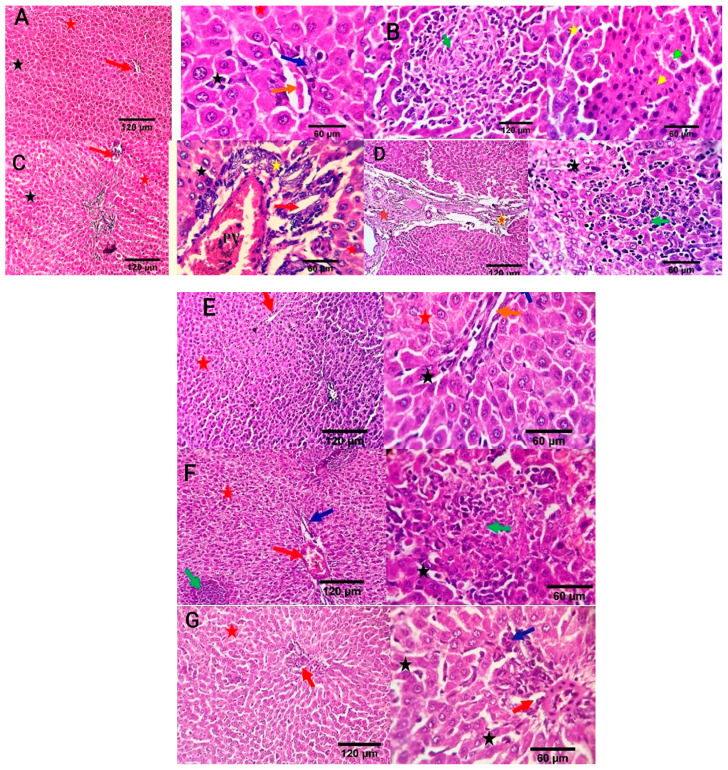
Photomicrographs of liver sections of rats using H&E staining at 7th day post infection with *S*. Typhimurium. (**A**) noninfected and nontreated rats showing a normal central vein and portal area comprising vascular and biliary structures (red arrow at 120 µm; orange and blue arrows at 60 µm) and, hepatic sinusoids (black star), and hepatocytes (red star). (**B**) infected non treated rats showing focal hepato-cellular degeneration (green arrowhead), early necrotic changes (yellow arrowhead), and scattered apoptosis (yellow star) beside focal round cells aggregation (dark green arrow). (**C**) rats were infected and treated with 45 mg/kg CIP (ciprofloxacin) orally once daily for 5 days, showing mildly congested portal blood vessels and biliary proliferation (yellow star and red arrow). Normal hepatic sinusoids (black star) and hepatocytes (red star) are also seen. (**D**) rats were infected and received CIP+OB-LNPs (45 mg/kg bw + 2.5 mL/kg bw) showing apparently normal hepatic parenchyma with occasional portal fibroplasia (brown star), biliary proliferation (orange star), reactive Von-Kupffer cells (black star), and focal interstitial round cells aggregation (dark green arrow). (**E**) rats were infected and treated with CIP+ LS-LNPs (45 mg/kg bw + 2.5 mL/kg bw), showing normal central vein (red arrow), portal area comprising vascular and biliary structures (orange and blue arrows), hepatic sinusoids (black star), and hepatocytes (red star). (**F**) rats were infected and treated with OB-LNPs (2.5 mL/kg bw), showing apparently normal hepatic parenchyma (red arrow, red star, and dark blue arrows) with occasional reactive Von-Kupffer cells (black star) and focal interstitial round cells aggregation (dark green arrow). (**G**) rats were infected and treated with LS-LNPs (2.5 mL/kg bw) daily for 5 days showing apparently normal hepatic parenchyma (red arrow, red star, and dark blue arrows) with occasional reactive Von-Kupffer cells (black star).

Liver sections in the negative control group revealed a normal central vein, a portal area comprising vascular and biliary structures, hepatic sinusoids, and hepatocytes (Figure 7A). In the positive control group, liver sections showed portal vascular congestion, leucocytic infiltration, sinusoidal dilatation, biliary proliferation, hepatocellular atrophy, disorganization, and scattered apoptosis (Figure 7B). The Ciprofloxacin-treated group showed normal hepatic sinusoids and hepatocytes, mildly congested portal blood vessels, and biliary proliferation (Figure 7C). The CIP+OB-LNPs treated group showed normal portal blood vessels, hepatic sinusoids, and hepatocytes, as well as mild portal biliary proliferation (Figure 7D). Likely, CIP + LS-LNPs treated group showed mild portal biliary proliferation (Figure 7E). Portal vascular congestion, mild biliary proliferation, and portal lymphocytic aggregation were detected in the OB-LNPs treated group (Figure 7F). The LS-LNPs treated group showed normal portal structures, hepatocytes, central vein, and sinusoids (Figure 7G).

### 3.7. In Vivo Antifungal Activity of LC-LNPs and OB-LNPs against T. rubrum

**Figure 8:**
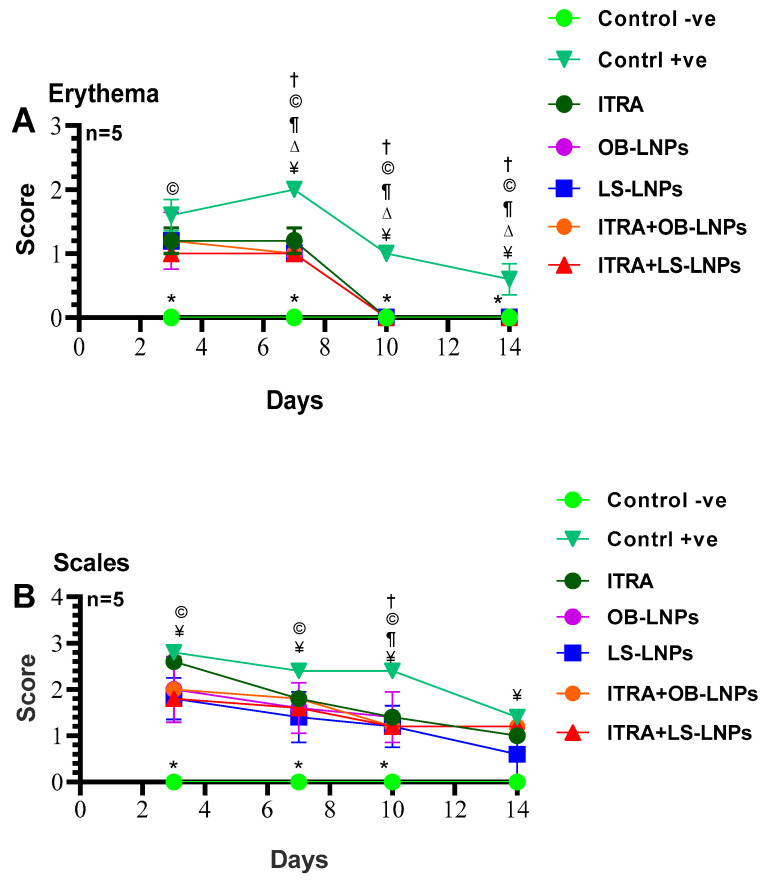

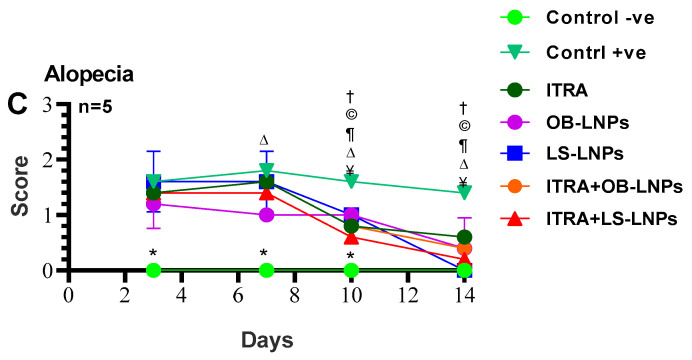
Means of erythema (**A**), scales (**B**), and alopecia (**C**) scores in *Trichophyton rubrum* infected guinea pigs in different groups. Symbols on the graphs indicate significant differences among the treated groups (itraconazole (ITRA), ITRA+LC-LNPs, ITRA+OB-LNPs, OB-LNPs, and LS-LNPs) and the untreated positive control group (*p* < 0.05). *p*-value was estimated using Exact Wilcoxon test.

As shown in Figures S1, S2 and Figure 8, the untreated control positive group revealed a clear lesion of erythema, scales, and alopecia with elevated lesion scores. All treated groups showed a reduction in lesion scores at 14 days PT. Meanwhile, complete elimination of lesion scores occurred on the 28th day of PT in all treated groups (Table 3).

On day 14 PT, all treated animals were culture-negative, but some animals remained microscopy positive (Table 3). All treated animals were culture- and microscopy-negative on day 28th PT. The mycological cure rate was 80% (samples from 4/5 animals in the group were microscopy and culture negative for successive two weeks) in the ITRA+LS-LNPs (G4) and ITRA+OBLNPs treated groups (G5) after 14 days of treatment.

## 4. Discussion

Nanotechnology plays a vital role in providing effective antimicrobial nanotherapeutics to counteract antimicrobial resistance [53,54]. In addition, lignin NPs are a great carrier for hydrophobic molecules [8], and *L. siceraria* and *O. basilicum* extracts are known to be natural stabilizing and reducing substances for the formation of NPs with low toxicity and eco-friendliness [55]. This study investigated for the first time the antimicrobial and anti-efflux activities of both OB-LNPs and LS-LNPs as green NPs. The phytochemical profiling of *O. basilicum* and *L. siceraria* seed oil that was described in phytochemical studies [12,13,14,15,18,19] potentially responsible for the antimicrobial and antioxidant properties of both extracts. The previously described [12] phytochemical profiling of *L. siceraria* seed oil indicated an acid value of 10.77± 0.06 mg/g, free fatty acids 2.23 ± 0.04 mg/g, a peroxide value of 0.55 ± 0.04 mEq/kg, a saponification value of 71.52 ± 0.03 mg/g, and an ester value of 60.75 ± 0.03 mg/g. Gas chromatography–mass spectrometry (GC–MS) spectral analysis showed several fatty acids such as stearolic acid, palmitic acid, erucic acid, stearic acid, and other active ingredients. The GC-MS phytochemical profiling of *O. basilicum* extracts revealed the presence of 75 components, including hydrocarbons, monoterpenes, triterpenes, sequiterpenes, phthalates, and phytosterols [15]. The main detected fatty acids were terpineol (1.42%), linalool (7.65%), tau-cadinol (13.55%), methyl palmitate (14.24%), palmitic acid (14.31%), linolenic acid (1.30%), and methyl linolinate (17.72%) [15]. The sub-MICs of OB-LNPs and LS-LNPs diminished efflux pump activity and downregulated the efflux pump genes (*ram*A and *acr*B) of *Salmonella enterica* strains, inducing antimicrobial activity. This agrees with Khosravani et al. [56], who documented the inhibitory effect of *Artemisia tournefortina* extract on the *acr*B gene of *S. enteritidis* strains after treatment with a sub-MIC concentration of the extract. Moreover, Mehta and Jandaik [57] and Mahmood et al. [58] reported the inhibitory activity of *Phyllanthus emblica* extract and zinc oxide NPs on the efflux pumps of *S*. Typhimurium strains. Moreover, Zhang et al. [59] found that pyrano-pyridine (MBX2319) is an efflux pump inhibitor against resistance nodulation diffusion (RND) of the efflux system in *Enterobacteriaceae*. It could reduce the MICs of ciprofloxacin against *E. coli* strains.

In vivo testing of LS-LNPs for treating *S*. Typhimurium infection in a rat model indicated a significant decrease (*p* < 0.05) in biochemical blood parameters, including AST, ALT, TP, and Alb, in comparison with the infected, non-treated group. The hepatoprotective effect is attributed to its flavonoids and phenolic content [60]. Nevertheless, treatment with OB-LNPs alone or in combination with ciprofloxacin induced a significant decrease (*p* < 0.05) in liver enzymes. The current findings showed a significant increase in total protein in all treated groups compared to the infected non treated group on 7th and 14th day PI.

Albumin level is considered an indicator of liver function [61]. Our results revealed a significant increase in the serum albumin level in all treated groups when compared to the non-treated group on both 7th and 14th day PI. However, silver nanoparticles (AgNPs) reduced serum albumin levels due to the stress effect on the hepatic tissue [61]. Significant improvement was observed in urea and creatinine levels in all treated groups when compared to the non-treated group on both 7th and 14th day PI. While Ramadan et al. [62] reported that oral administration of zinc oxide NPs to rats at different doses induced a significant increase in urea and creatinine levels.

The serum LDH level has been reported to be an indicator of tissue damage [63]. There was a significant decrease (*p* < 0.05) in the concentration of LDH in the CIP+LS-LNPs-treated group compared to the non-treated group, indicating a treatment effect on the hepatic tissue. Similarly, Singh et al. illustrated that *L. siceraria* fruit powder extract showed cardioprotective effects in both isoprenaline and doxorubicin-induced cardiotoxicity, as indicated by the significant decrease in creatine-kinase, lactate dehydrogenase, and low-density lipoprotein [64]. These effects are attributed to the elevated concentration of polyphenolic components in fruits [65].

Concerning the antioxidant effect of nanocomposites-treated groups, there was a significant decrease in the serum MDA concentration in all treated groups compared to the non-treated group on both 7th and 14th day PI. These antioxidant and lipid peroxidation effects of *L. siceraria* seeds are due to the presence of bioactive compounds such as flavonoids, phenols, tannins, and terpenoids [66]. Furthermore, lignin possesses a free radical scavenging activity that reduces oxygen radicals and stabilizes oxidation reactions owing to the phenolic hydroxyl groups in its structure [10]. A significant increase in the serum TAC level was detected in all treated groups compared with the non-treated group. These results go hand in hand with those of Karakas and Hacioglu Dogru [67], who found that AgNPs produced from *O. basilicum* leaf extract possess greater antioxidant activity than those produced from *O. basilicum* extract. Moreover, the antioxidant activity of *O. basilicum* hydroethanolic extract raised the serum levels of antioxidant and oxidant markers in rats [68]. *L. siceraria* seeds extract (LSSE) elevated the antioxidant enzymes owing to its content of phytochemicals, which act as reducing agents, metal chelators, singlet oxygen quenchers, and hydrogen donors [69,70,71,72].

A significant increase in haematological parameters, including Hb, was observed in CIP, OB-LNPs, and LS-LNPs treated groups compared to the non-treated group on both the 7th and 14th days of PI.

Interestingly, a significant decrease in total leukocyte count and neutrophil count, in addition to an increase in lymphocyte count, was observed in all treated groups in comparison with the non-treated group on both the 7th and 14th day PI. However, the total leukocytic count and neutrophils increased in mice treated with fresh *L. siceraria* fruit juice after induction of immunosuppression by pyrogallol [73]. Regarding the activity of the immune system, a significant elevation in macrophage phagocytic activity was observed in the OB-LNPs and LS-LNPs-treated groups in comparison with the non-treated group on both the 7th and 14th days of PI.

*Salmonella* infection stimulates proinflammatory cytokines such as IFN-γ and TNF-α in both infected and adjacent cells [74]. OB-LNPs and LS-LNPs-treated groups, either alone or in combination with ciprofloxacin, showed a decrease in IFN-γ and TNF-α levels on both the 7th and 14th days of PI.

*Salmonella* load in the liver, spleen, and intestine homogenates was high in both the 3rd and 7th days of PI, while the bacterial count decreased to zero in the 14th day of PI in all treated groups compared with the non-treated group. A significant decrease in bacterial count was observed in the combined treatment groups (OB-LNPs or LS-LNPs + CIP) compared to the other treated groups. Moreover, *Salmonella* load in the 7th day PI (4 log_10_ to 4.7 log_10_ CFU/g), was less than bacterial load in the 3rd day PI (4.5 log_10_ to 4.9 log_10_ CFU/g), while no growth was detected at day 14 PI.

Histopathological analysis of the intestinal and liver tissues supported these results. In the non-treated group, *S.* Typhimurium infection was reflected in the histopathological findings through diffuse inflammatory cell infiltration and desquamation of the epithelial tissue of the intestine. In addition, leucocytic infiltration, sinusoidal dilatation, hepatocellular atrophy, disorganization, and scattered apoptosis were observed in liver tissues. Meanwhile, the administration of CIP in combination with LS-LNPs and OB-LNPs resulted in a reduction in both intestinal inflammation and liver damage, returning their tissues to normal.

Dermatophytosis of the skin is mainly caused by the *Trichophyton* species. *T*. *rubrum* and *T*. *mentagrophytes* have been documented as the primary pathogens that induce onychomycosis and tinea corporis [75]. *T. rubrum* is well-known for its fungal resistance [76]. This study investigated, for the first time, an alternative topical treatment for *T. rubrum* infection using LS-LNPs and OB-LNPs. In vitro testing of both LS-LNPs and OB-LNPs against *T. rubrum* using the agar well diffusion method and broth microdilution test indicated a significant inhibition of fungal growth with an inhibition zone of 22–24 mm and MIC values ranging from 0.125 to 2 µg/mL. It has been reported that *L. siceraria* seed extract has antifungal potential against *Candida* species and *Aspergillus niger* [13]. Interestingly, lignin NPs possess antifungal properties against filamentous fungi [5,77]. The in vivo therapeutic effects of LS-LNPs and OB-LNPs were evaluated against dermatophytosis induced in a guinea pig model. Significant differences in lesion scores were found between groups treated with Itra+LS-LNPs and Itra+OB-LNPs and the positive control group after 10 days of treatment. In addition, significant differences were found in the direct microscopy and culture results of hair and scale samples from all treated groups when compared with the positive control group after 14 days PT. Fortunately, most treated animals were culture-negative, but some animals remained microscopically positive on day 14 PT (Table 2). This may be attributed to the presence of non-viable conidia and hyphae that were indicated microscopically and would not grow on culture [78]. Skin redness, scales, and alopecia were significantly diminished following the topical application of LS-LNPs and OB-LNPs alone or in combination with itraconazole for 12 days. The efficacy of treatment was determined by hair growth recurrence in the infected areas in all treated groups compared to the infected non-treated group. Owing to the small size of lignin NPs, keep them in direct contact with the stratum corneum and facilitate the entry of drugs into the skin [79,80]. Most treated animals were culture-negative, but some animals remained microscopy-positive on day 14 PT. This may be attributed to the presence of non-viable conidia and hyphae that were indicated microscopically and would not grow on culture [78]. The mycological cure rate in *T*. *rubrum* infected and treated animals after 14 days of PT was 80%. Similar studies documented that the cure rates after treatment with antifungal agents ranged from 80 to 100% [78,81].

## 5. Conclusions

In essence, OB-LNPs and LS-LNPs are natural efflux inhibitors that can enhance the activity of ciprofloxacin by suppressing the resistance mechanism of *Salmonella enterica* strains to this antibiotic and reducing the chance of resistance development when combined with antibiotics. In addition, OB-LNPs and LS-LNPs alone or in combination with itraconazole had a high inhibitory effect against *T. rubrum* infection in a guinea pig model with reduced clinical symptoms within 12 days. Moreover, OB-LNPs and LS-LNPs possess immunomodulatory and antioxidant potential. Hence, lignin is a naturally derived source of nanoparticles to be used as a drug delivery system for *L. siceraria* and *O. basilicum* against Salmonellosis and dermatophytosis in an attempt to tackle the looming crisis of antimicrobial resistance, paving the way for further investigations in this aspect. This study opens up new horizons for future studies to explore ciprofloxacin or itraconazole co-loaded with the extracts in the lignin nanoparticles.

## Figures and Tables

**Table 1:** The antimicrobial activity of LS-LNPs and OB-LNPs.

Species (No. of Strains)	LS-LNPs MIC Range (µg/mL)	OB-LNPs MIC Range (µg/mL)
*Salmonella enterica* (10)	0.5–4	0.5–2
*S. aureus* (10)	8–16	2–16
*K. pneumoniae* (10)	4–16	2–16
*E. faecalis* (10)	4–16	2–16
*E. coli* (10)	4–16	4–16
*T. rubrum* (3)	0.125–1	0.25–2
*T. mentagrophytes* (4)	2–8	4–16
*M. canis* (3)	4–8	8–16

**Table 2:** Effects of LS-LNPs, OB-LNPs, and ciprofloxacin on haematological, biochemical, oxidative stress mediators and the immunological parameters of rats on 7- and 14-days post-challenge with *S*. Typhimurium.

Items	At 7 Days Post Infection									At 14 Days Post Infection								
	NC	PC	CIP	CIP + OB-LNPs	CIP + LS-LNPs	OB-LNPs	LS-LNPs	±SE	*p*-Value	NC	PC	CIP	CIP + OB-LNPs	CIP + LS-LNPs	OB-LNPs	LS-LNPs	±SE	*p*-Value
ALT	36.40 ^d^	71.80 ^a^	54.80 ^b^	52.20 ^b,c^	48.40 ^c^	50.60 ^b,c^	49.20 ^b,c^	3.98	<0.0001	21.13 ^d^	48.53 ^a^	41.17 ^b^	37.89 ^b,c^	34.79 ^c^	37.45 ^b,c^	41.94 ^b^	3.20	<0.0001
AST	89.60 ^b^	113.32 ^a^	89.60 ^b^	89.16 ^b^	75.32 ^c^	89.12 ^b^	89.22 ^b^	4.25	<0.0001	67.74 ^bc^	91.20 ^a^	71.72 ^b,c^	65.24 ^c^	67.94 ^b,c^	70.56 ^b,c^	74.84 ^b^	3.29	<0.0001
Creatinine	0.37 ^d^	0.74 ^a^	0.54 ^b,c^	0.51 ^c^	0.55 ^b,c^	0.55 ^b,c^	0.61 ^c^	0.04	<0.0001	0.46 ^e^	0.69 ^a^	0.60 ^b^	0.58 ^b^	0.55 ^b,c^	0.52 ^c,d^	0.47 ^d,e^	0.03	<0.0001
Urea	37.45 ^d,e^	55.38 ^a^	42.86 ^c,d^	41.38 ^c,d,e^	34.35 ^e^	44.82 ^b,c^	50.10 ^a,b^	2.72	<0.0001	29.40 ^b^	34.30 ^a^	30.56 ^b^	24.12 ^c^	24.22 ^c^	29.70 ^b^	29.46 ^b^	1.36	<0.0001
LDH	413.60 ^d^	548.80 ^a^	511.80 ^a,b^	523.40 ^a,b^	454.80 ^c^	498.00 ^b^	482.00 ^b,c^	15.08	<0.0001	468.60 ^b^	553.20 ^a^	520.20 ^a,b^	510.00 ^a,b^	394.20 ^c^	545.80 ^a^	503.60 ^a,b^	13.4	<0.0001
TP	6.90 ^a^	5.98 ^c^	6.67 ^ab^	6.42 ^b^	6.68 ^a,b^	6.40 ^b^	6.51 ^a,b^	0.11	0.0030	7.05 ^a,b^	6.29 ^e^	6.72 ^b,c,d^	6.63 ^c,d,e^	7.10 ^a^	6.47 ^d,e^	6.89 ^a,b,c^	0.11	0.0002
Alb	3.50 ^a,b^	3.19 ^c^	3.51 ^ab^	3.49 ^ab^	3.57 ^a^	3.35 ^b^	3.45 ^a,b^	0.05	0.0008	3.72 ^a^	3.41 ^b^	3.58 ^a,b^	3.60 ^a,b^	3.64 ^a^	3.60 ^a,b^	3.53 ^a,b^	0.04	0.0405
MDA	133.40 ^c,d^	199.00 ^a^	159.60 ^b^	170.40 ^b^	134.60 ^c,d^	150.40 ^b,c^	125.00 ^d^	9.73	<0.0001	119.36 ^b^	139.26 ^a^	133.76 ^a^	120.40 ^b^	118.94 ^b^	136.62 ^a^	143.10 ^a^	3.91	<0.0001
TAC	235.20 ^a^	161.00 ^c^	190.80 ^b^	191.20 ^b^	191.60 ^b^	190.40 ^b^	179.80 ^b,c^	8.42	0.0001	179.60 ^a^	142.40 ^c^	155.60 ^b,c^	167.80 ^a,b^	180.80 ^a^	172.80 ^a,b^	168.60 ^a,b^	5.17	0.0005
Hb	11.38 ^a^	9.78 ^c^	10.62 ^b^	10.68 ^b^	10.67 ^b^	10.56 ^b^	10.78 ^b^	0.18	<0.0001	12.37 ^a,b^	10.32 ^c^	11.40 ^b,c^	12.12 ^a,b^	12.53 ^a,b^	11.96 ^a,b^	12.73 ^a^	0.31	0.0044
WBCs	1.94 ^c^	3.08 ^a^	2.41 ^bc^	2.41 ^bc^	2.12 ^b,c^	2.52 ^b^	2.51 ^b^	0.14	0.0007	2.38 ^c^	2.98 ^a^	2.70 ^a,b,c^	2.78 ^a,b^	2.62 ^b,c^	2.70 ^a,b,c^	2.64 ^a,b,c^	0.07	0.0349
Neutrophil	21.80 ^c^	40.80 ^a^	34.20 ^b^	34.60 ^b^	33.60 ^b^	35.80 ^b^	32.60 ^b^	2.17	<0.0001	19.80 ^c^	30.40 ^a^	28.40 ^ab^	26.40 ^b^	25.00 ^b^	28.20 ^a,b^	27.60 ^a,b^	1.29	<0.0001
Lymphocytes	78.20 ^a^	57.60 ^c^	66.20 ^b^	66.00 ^b^	66.40 ^b^	63.40 ^b^	66.00 ^b^	2.32	<0.0001	79.80 ^a^	67.80 ^c^	69.40 ^bc^	71.20 ^b,c^	72.0 ^b^	68.80 ^b,c^	70.00 ^b,c^	1.52	<0.0001
Phagocytosis	31.60 ^a^	19.00 ^e^	22.00 ^c,d,e^	24.60 ^bc^	26.60 ^b^	21.40 ^d,e^	22.80 ^c,d^	1.56	<0.0001	36.60 ^a^	26.60 ^d^	30.00 ^c^	32.60 ^b^	34.00 ^b^	26.80 ^d^	27.80 ^c,d^	1.47	<0.0001
PI	1.34 ^a^	1.05 ^b^	1.11 ^b^	1.12 ^b^	1.13 ^b^	1.10 ^b^	1.10 ^b^	0.04	0.0041	1.23 ^a^	1.11 ^c^	1.15 ^bc^	1.14 ^b,c^	1.17 ^b^	1.13 ^b,c^	1.14 ^b,c^	0.01	0.0002
INF-γ	35.68 ^e^	52.42 ^a^	41.64 ^cd^	40.50 ^cd^	38.56 ^d,e^	46.30 ^b^	44.44 ^b,c^	2.09	<0.0001	32.70 ^c^	49.94 ^a^	39.18 ^b^	35.72 ^b,c^	38.44 ^b,c^	41.74 ^b^	40.54 ^b^	2.05	0.0001
TNF-α	26.46 ^e^	48.64 ^a^	40.54 ^c^	42.86 ^bc^	47.30 ^a,b^	32.94 ^d^	35.52 ^d^	3.03	<0.0001	28.06 ^d^	53.32 ^a^	44.90 ^a^	49.22 ^a^	51.56 ^a^	40.42 ^c^	44.56 ^b,c^	3.23	<0.0001

The mean values followed by different superscript letters ^a,b,c,d,e^ in the same row are significantly different (*p* < 0.05). SE: standard error of mean; NC (negative control): noninfected nontreated rats; PC (Positive Control): rats were infected with *S*. *typhimurium* intraperitonally; CIP (ciprofloxacin): rats were infected with *S*. Typhimurium intraperitonally and treated with 45 mg/kg CIP orally once daily for 5 days; CIP-OB-LNPs: rats infected with *S*. Typhimurium intraperitonally and received Cip+ *Ocimum Basilicum* loaded-Lignin Nanoparticles (45 mg/kg bw + 2.5 mL/kg bw), Cip+ LS-LNPs: rats were infected with *S*. Typhimurium intraperitonally and treated with Cip+ *Lagenaria Siceraria* loaded-Lignin Nanoparticles (45 mg/kg bw + 2.5 mL/kg bw); OB-LNPs: rats were infected with *S.* Typhimurium intraperitonally and treated with *Ocimum Basilicum* loaded-Lignin Nanoparticles (2.5 mL/kg bw); LS-LNPs: rats were infected with *S*. Typhimurium intraperitonally and treated with *Lagenaria Siceraria* loaded-Lignin Nanoparticles (2.5 mL/kg bw), as a single dose daily for 5 days.

**Table 3:** Mycological evaluation of dermatophytosis treatment in a guinea pig model.

	3rd Day PT							7th Day PT							10th Day PT							14th Day PT							28th Day PT						
	G1	G2	G3	G4	G5	G6	G7	G1	G2	G3	G4	G5	G6	G7	G1	G2	G3	G4	G5	G6	G7	G1	G2	G3	G4	G5	G6	G7	G1	G2	G3	G4	G5	G6	G7
M	0	4	3	2	3	3	3	0	5	3	3	3	4	4	0	4	3	2	3	2	2	0	3	2	1	1	2	2	0	0	0	0	0	0	0
C	0	4	2	2	2	2	5	0	3	2	1	1	2	2	0	3	2	0 *	0 *	1	1	0	3	0 *	0 *	0 *	0 *	0 *	0	0	0	0	0	0	0
M&C	0	3	2	2	2	2	3	0	3	2	1	1	2	2	0	3	2	0 *	0 *	1	1	0	3	0 *	0 *	0 *	0 *	0 *	0	0	0	0	0	0	0

M: indicates No. of microscopy positive; C: No. of culture positive animals in each group (5 guinea pigs/group). Treated groups versus positive controls were compared using the fisher’s exact test; * Differs significantly with positive control *p* < 0.05. G1: non infected control negative group; G2: infected untreated control group; G3: infected and treated with 10 mg/kg itraconazole daily by oral gavage; G4: infected and treated group with *Lagenaria siceraria* loaded lignin nanoparticles + itraconazole; G5: infected and treated group with *Ocimum basilicum* loaded lignin nanoparticles + itraconazole; G6: infected and treated group with *Ocimum basilicum* loaded lignin nanoparticles; G7: infected and treated group with *Lagenaria siceraria* loaded lignin nanoparticles.

## Data Availability

The datasets generated or analyzed during the current study are available from the corresponding author upon reasonable request.

## Supplementary Materials

The following supporting information can be downloaded at: https://www.mdpi.com/article/10.3390/antiox13070865/s1, Table S1: Oligonucleotide primer sequences used in the study [82,83,84,85,86,87]; Table S2: Antibacterial and antifungal activities of OB-LNPs and LS-LNPs; Table S3: Effects of OB-LNPs and LS-LNPs on efflux pump activity and expression of *ram*A and *acr*B genes in ciprofloxacin-resistant *Salmonella enterica* strains. Figure S1: *Trichophyton rubrum*-infected guinea pig models at day 3 post treatment G1: infected untreated control positive group; G2: infected and treated with 10 mg/kg itraconazole once daily through oral gavage; G3: Itra+LS-LNPs treated group; G4: Itra+OB-LNPs treated group; G5: OB-LNPs; G6: LS-LNPs treated groups topically once daily. Figure S2: *Trichophyton rubrum*-infected guinea pig models at day 14 post-treatment G1: infected untreated control positive group; G2: infected and treated with 10 mg/kg itraconazole once daily through oral gavage; G3: Itra+LS-LNPs treated group; G4: Itra+OB-LNPs treated group; G5: OB-LNPs; G6: LS-LNPs treated groups topically once daily.
