# Supplementary material for: Ocimum basilicum and Lagenaria siceraria Loaded Lignin Nanoparticles as Versatile Antioxidant, Immune Modulatory, Anti-Efflux, and Antimicrobial Agents for Combating Multidrug-Resistant Bacteria and Fungi
Source: Antioxidants (Basel). 2024 Jul 19;13(7):865. doi: 10.3390/antiox13070865 (PMC11273778; doi:10.3390/antiox13070865)
Supplement: Supplementary file 1 [file antioxidants-13-00865-s001.zip › Supplementary Figures.pdf]

Supplementary Figure legends

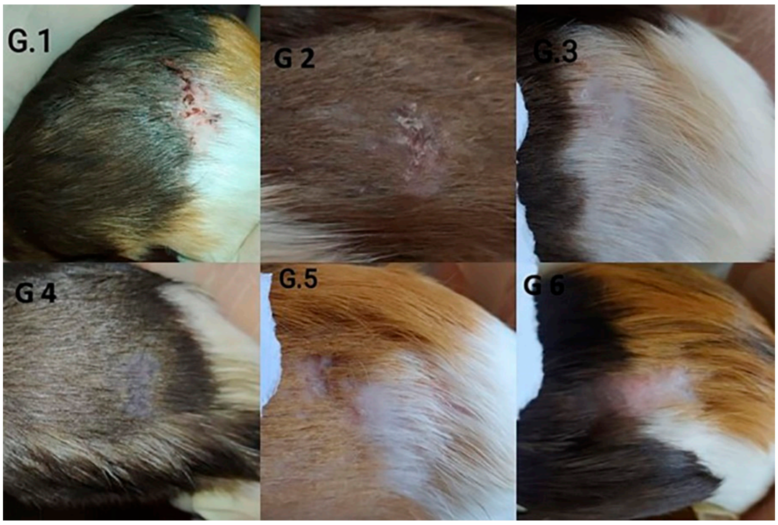

Figure S1: *Trichophyton rubrum* infected guinea pig models at day 3 post treatment G1: infected untreated control positive group; G2: infected and treated with10 mg/kg itraconazole once daily through oral gavage; G3: Itra+LS-LNPs treated group; G4: Itra+OB-LNPs treated group; G5: OB-LNPs; G6: LS-LNPs treated groups topically once daily.

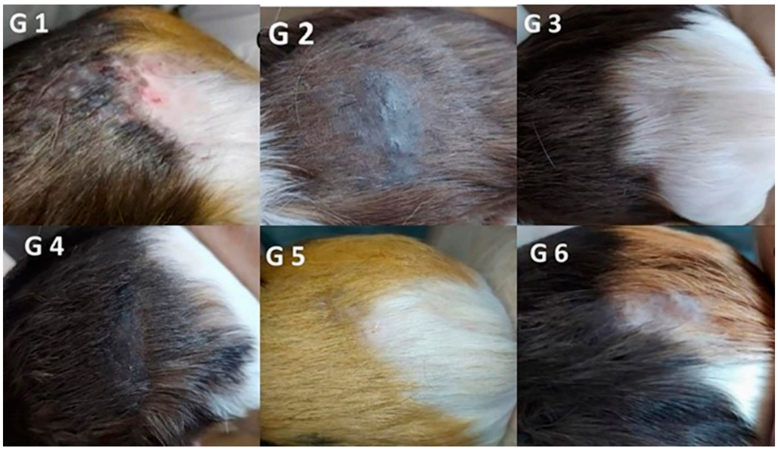

Figure S2: *Trichophyton rubrum* infected guinea pig models at day 14 post treatment G1: infected untreated control positive group; G2: infected and treated with 10 mg/kg itraconazole once daily through oral gavage; G3: Itra+LS-LNPs treated group; G4: Itra+OB-LNPs treated group; G5: OB-LNPs; G6: LS-LNPs treated groups topically once daily.
