# Supplementary material for: Ocimum basilicum and Lagenaria siceraria Loaded Lignin Nanoparticles as Versatile Antioxidant, Immune Modulatory, Anti-Efflux, and Antimicrobial Agents for Combating Multidrug-Resistant Bacteria and Fungi
Source: Antioxidants (Basel). 2024 Jul 19;13(7):865. doi: 10.3390/antiox13070865 (PMC11273778; doi:10.3390/antiox13070865)
Supplement: Supplementary file 1 [file antioxidants-13-00865-s001.zip › Supplementary Table S1.pdf]

Table S1: Oligonucleotide primer sequences used in the study

Target gene	Nucleotide sequence (5′→3′)	Gene accession no	Annealing temperature (°C)	Amplicon size (bp)	References
S. aureus Sa0836	F:TCGAAATTAAATGTTGTCGTGTCTTC<br>R: TCATTTTTGACATGRAGAGAAACATC	NC_002745.2	55	573	[82]
E. faecalis ddl	F: ATCAAGTACAGTTAGTCT<br>R: ACGATTCAAAGCTAACTG	NC_019770.1	54	941	[83]
E. coli 16S rRNA	F: GACCTCGGTTTAGTTCACAGA<br>R: CACACGCTGACGCTGACCA	NC_000913.3	56	585	[84]
K. pneumoniae 16S-23S ITS	F: ATTTGAAGAGGTTGCAAACGAT<br>R: TTCACTCTGAAGTTTTCTTGTGTTC	NR_119278.1	55	130	[85]
Salmonella enterica invA	F: GTGAAATTATCGCCACGTTCGGGCA<br>R: TCATCGCACCGTCAAAGGAACC	NC_003197.2	50	284	[86]
Dermatophytes ITS1, ITS2, 5.8S rDNA	F: TCCGTAGGTGAACCTGCGG<br>R: TCCTCCGCTTATTGATATGC	NC_001144.5	56	680-700	[87]

ITS, Internal transcribed spacer; F, forward; R, reverse; bp, base pair.

References

82. Liu, D.; Lawrence, M.L.; Austin, F.W. Evaluation of PCR primers from putative transcriptional regulator genes for identification of *Staphylococcus aureus*. *Lett Appl Microbiol.* **2005**; *40*: 69–73.

83. Dutka-Malen, S.; Evers, S.; Courvalin, P. Detection of glycopeptide resistance genotypes and identification to the species level of clinically relevant enterococci by PCR. *J Clin Microbiol.* **1995**; *33*:24–27.

84. Amit-Romach, E.; Sklan, D.; Uni, Z. Microflora Ecology of the Chicken Intestine Using 16S Ribosomal DNA Primers. *Poultry Science,* **2004**; 83**,** 1093-1098.

85. Liu, Y.; Liu, C.; Zheng, W.; Zhang, X.; Yu, J.; Gao, Q. Huo; Y.; Huang, X. PCR Detection of *Klebsiella pneumoniae* in Infant Formula Based on 16S-23S Internal Transcribed Spacer. *Int. J. Food Microbiol.* **2008**, *125*, 230–235.

86. De Clercq, D.; Ceustermans, A.; Heyndrickx, M.J.; Coosemans, J.; Ryckeboer, A. A rapid monitoring assay for the detection of *Salmonella* spp. and *Salmonella* Senftenberg strain W775 in composts. *J Appl Microbiol*. **2007**, *103*, 1364-5072.

87. White, T.J.; Bruns, T.D.; Lee, S.B.; Taylor, J.W. Amplification and direct sequencing of fungal ribosomal RNA genes for phylogenetics. In *PCR Protocols: A Guide to Methods and Applications*; Innis, M.A., Celfand, D.H., Sninsky, J.J., White, T.J., Eds.; Academic Press: San Diego, CA, USA, 1990; pp. 315–322.
