# Supplementary material for: Ocimum basilicum and Lagenaria siceraria Loaded Lignin Nanoparticles as Versatile Antioxidant, Immune Modulatory, Anti-Efflux, and Antimicrobial Agents for Combating Multidrug-Resistant Bacteria and Fungi
Source: Antioxidants (Basel). 2024 Jul 19;13(7):865. doi: 10.3390/antiox13070865 (PMC11273778; doi:10.3390/antiox13070865)
Supplement: Supplementary file 1 [file antioxidants-13-00865-s001.zip › Supplementary Table S3.pdf]

Table S3: Effects of OB-LNPs and LS-LNPs on efflux pump activity and expression of *ram*A and *acr*B genes in ciprofloxacin-resistant *Salmonella enterica* strains

**Strains**	**CIP MIC (µg/mL)**	**Efflux pump activity**		**OB-LNPs**					**LS-LNPs**				
		MC_{EtBr} (µg/mL)	Efflux index	MIC	Anti-efflux pump activity		Fold change		MIC (µg/mL)	Anti-efflux pump activity		Fold change	
					MC_{EtBr} (µg/mL)	Efflux index*	*ram*A	*acr*B		MC_{EtBr} (µg/mL)	Efflux index*	*ram*A	*acr*B
S. Infantis	4	1	3	0.5	0.25	1	0.3789	0.4796	0.5	0.5	1	0.4730	0.6878
S. Typhemurium	32	1.5	5	1	0.5	1	0.5434	0.6199	2	0.5	1	0.7738	0.8351
S. Magherofelt	32	2	7	1	1	3	0.3143	0.5105	2	1.5	5	0.5548	0.6373
S. Typhemurium	16	2	7	0.5	0.5	1	0.2989	0.3842	1	0.5	1	0.4601	0.5035

OB-LNPs: Ocimum basilicum loaded lignin nanoparticles, LS-LNPs: *Lagenaria siceraria* loaded lignin nanoparticles, CIP: Ciprofloxacin.

*Reference strain assigned MC_{EtBr} value of 0.25 µg/mL.
